# Protective Effect of Oligonol on Dimethylnitrosamine-Induced Liver Fibrosis in Rats via the JNK/NF-κB and PI3K/Akt/Nrf2 Signaling Pathways

**DOI:** 10.3390/antiox10030366

**Published:** 2021-02-28

**Authors:** Changyong Lee, Jeonghyeon Bak, Sik Yoon, Jeon-Ok Moon

**Affiliations:** 1 College of Pharmacy, Pusan National University, Busan 46241, Korea; qhrrn79@pusan.ac.kr (C.L.); jayla3434@gmail.com (J.B.); 2 Department of Anatomy, College of Medicine, Pusan National University, Yangsan 50612, Korea; sikyoon@pusan.ac.kr

**Keywords:** oligonol, anti-oxidative, anti-inflammatory, hepatoprotective, anti-fibrotic, NF-κB, Nrf2 signaling pathway

## Abstract

Oligonol is a low molecular weight polyphenol product derived from lychee fruit by a manufacturing process. We investigated oligonol’s anti-fibrotic effect and the underlying mechanism in dimethylnitrosamine (DMN)-induced chronic liver damage in male Sprague–Dawley rats. Oral administration of oligonol (10 and 20 mg/kg body weight) ameliorated the DMN-induced abnormalities in liver histology and serum parameters in rats. Oligonol prevented the DMN-induced elevations of TNF-α, IL-1β, IL-6, cyclooxygenase-2, and inducible nitric oxide synthase expressions at the mRNA level. NF-κB activation and JNK phosphorylation in DMN-treated rats were ablated by oligonol. Oligonol reduced the enhanced production of hepatic malondialdehyde and reactive oxygen species and recovered protein SH, non-protein SH levels, and catalase activity in the DMN treated liver. Nrf2 translocation into the nucleus was enhanced, and PI3K and phosphorylated Akt levels were increased by administering oligonol. The level of hepatic fibrosis-related factors such as α-smooth muscle actin, transforming growth factor-β1, and type I collagen was reduced in rats treated with oligonol. Histology and immunohistochemistry analysis showed that the accumulation of collagen and activation of hepatic stellate cells (HSCs) in liver tissue were restored by oligonol treatment. Taken together, oligonol showed antioxidative, hepatoprotective, and anti-fibrotic effects via JNK/NF-κB and PI3K/Akt/Nrf2 signaling pathways in DMN-intoxicated rats. These results suggest that antioxidant oligonol is a potentially useful agent for the protection against chronic liver injury.

## 1. Introduction

The liver performs numerous critical functions in life. Many industrial chemicals, plant toxins, herbal remedies, dietary supplements, and drugs are hepatotoxins that lead to liver dysfunction. Hepatotoxicity is one of the most frequent causes of discontinuing drug candidates’ development and the withdrawal of drugs from the market. In the initial stages of inflammation, various resident liver cells such as hepatocytes, Kupffer cells, and platelets are activated and increase the production of reactive oxygen species (ROS) and cytokines such as tissue necrosis factor-α (TNF-α) [1]. These mediators activate quiescent hepatic stellate cells (HSCs) that are localized in the space of Disse, resulting in an abnormal quantity and composition of the extracellular matrix (ECM) [2]. Liver fibrosis occurs in response to chronic liver injury that overwhelms the capacity of the liver to repair. There are no effective drugs for liver fibrosis. Therefore, it is crucial to suppress hepatic inflammation in the early stages of liver diseases. 

Oxidative stress has been recognized as one of the significant causes of liver disease. In particular, lipid peroxidation may compromise the membranes’ integrity and cause liver damage [3,4]. It has been documented that many antioxidants protect hepatocytes from lipid peroxidation in various liver injury models induced by hepatotoxins such as carbon tetrachloride (CCl_4_) or dimethylnitrosamine (DMN) [5,6]. Therefore, suppressing oxidative stress and the lipid peroxidation by antioxidants might be a potential therapeutic intervention for preventing or treating hepatic injuries.

Many phytochemicals in dietary fruits, vegetables, and whole grains are known to have potent antioxidant activity [7]. However, their low bioavailability limits their application as dietary supplements in humans. Oligonol is a phenolic product derived from lychee fruit (*Litchi chinensis* Sonn.) extracts composed of catechin monomers, procyanidin dimers, and oligomers of proanthocyanidins [8]. The bioavailability of oligonol is improved by a manufactured process compared with high-molecular-weight proanthocyanidins of fruit and plant sources [9]. Supplements of antioxidant oligonol have shown a variety of physiological and biochemical effects in in vivo models, cognitive-enhancing effects in an animal model of Alzheimer disease [10], antidiabetic action in streptozotocin-induced pancreatic damage in rats [11], protective effect against muscle loss in type 2 diabetes mouse model [12], and preventive efficacy of relapse in dextran sulfate sodium-ulcerative colitis [13]. We reported that oligonol exerts a hepatoprotective effect on the CCl_4_-intoxicated acute liver injury in rats through the nuclear factor-kappa B (NF-κB) and the mitogen-activated protein kinases (MAPKs) signaling pathways [14]. Since an intercept of the early stage of hepatic inflammation is a promising therapeutic strategy to prevent progression into the final stage of liver inflammation such as liver fibrosis, liver cirrhosis, we evaluated oligonol’s effects on a chronic liver injury model in rats using DMN. 

In this research, we report that oligonol showed antioxidative, anti-inflammatory, hepatoprotective, and anti-fibrotic activity in the DMN-induced chronic liver injury in rats. The action mechanism of oligonol was investigated by exploring the levels of NF-κB, nuclear factor-E2-related factor (Nrf2), as well as pro-inflammatory cytokines and proteins, and fibrotic mediators.

## 2. Materials and Methods

### 2.1. Chemicals

Oligonol is a commercially available polyphenol mixture comprised of 16.0% monomers (catechin, epicatechin, epicatechin gallate, and epigallocatechin gallate), 13.9% dimers (procyanidin A1, A2, B1, and B2), and proanthocyanidin oligomers (Amino Up Chemical Co., Ltd, Sapporo, Japan) [8]. 

### 2.2. Animals and Induction of Liver Fibrosis with DMN

**Figure 1:**
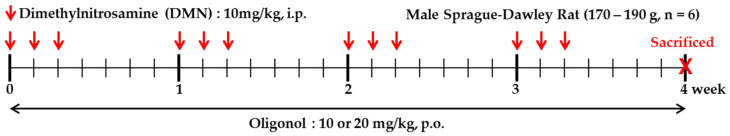
Treatment protocols for animal experiments.

Male Sprague–Dawley rats (Samtako, Osan, Korea) were given standard rat chow and free access to tap water with a 12 h light-dark cycle. With respect to ethical issues and scientific care, the animal protocol used in this study was reviewed and approved by the PNU-Institutional Animal Care and Use Committee (PNU-IACUC; Approval Number PNU-2016-1417). Twenty-four rats weighing 170–190 g and 6-7 weeks in age were divided into 4 groups (*n* = 6): Control, DMN, Oli10, and Oli20. Animals in the control received saline (DMN vehicle) by intraperitoneal (i.p.) injection and CMC (oligonol vehicle) by oral gavage; the DMN group received DMN and CMC, the Oli10 and Oli20 groups received DMN and oligonol at 10 and 20 mg/kg/day, respectively. Liver injury in the rats was induced by i.p. injection of 1% (*w*/*v*) DMN (10 mg/kg body weight) in saline once daily for three consecutive days per week for four weeks [6,15]. Oligonol was dissolved in a 0.5% CMC solution to a 10 mg/mL concentration administered once daily for four weeks by oral gavage (Figure 1). At the end of the fourth week, all rats were sacrificed immediately under anesthesia after an overnight fast. Blood samples for biochemical analyses were obtained from the inferior vena cava. The liver tissues were excised from these rats and frozen at liquid nitrogen before further analysis.

### 2.3. Histology

Histopathological preparations of the tissues were performed as described by our previous methods [15], stained with hematoxylin and eosin (H&E) and Sirius red to assess the architectural alteration and collagen accumulation. 

### 2.4. Biochemical Analysis of Serum Parameters

Serum aspartate transaminase (AST) and alanine transaminase (ALT) activities, albumin, and bilirubin levels were measured using commercial kits (Asan chemical Co., Cheonan, Korea) according to the manufacturer’s instructions. 

### 2.5. Malondialdehyde (MDA) Content

Liver tissue (0.1 g) was homogenized to determine MDA levels using 0.93 M trichloroacetic acid (TCA) solution composed of 26 mM thiobarbituric acid, 0.64 mM butylated hydroxytoluene, and 11 mM HCl. The homogenates were heated for 1 h and then centrifuged for 20 min at 2000× *g*. MDA concentration was calculated from the supernatant’s absorbance at 532 nm using MDA tetrabutylammonium as a standard [16]. 

### 2.6. Measurement of ROS Level

In the presence of esterase and ROS (∙O_2_^−^, ∙OH, and H_2_O_2_), nonfluorescent 2′,7′-dichlorofluorescin diacetate (DCFDA) was oxidized to the fluorescent 2′,7′-dichlorofluorescin. This fluorometric assay was applied to determine ROS level [17]. Changes in fluorescence intensity were measured with excitation and emission wavelengths set at 485 and 530 nm, respectively. 

### 2.7. Determination of Total Protein SH, Non-Protein SH Level, and Catalase Activity

For total SH measurement, samples were homogenized in 100 mM sodium phosphate buffer (pH 7.4). Homogenate (100 μL) was mixed with 100 μL 0.01 M 5,5-dithio-bis-2-nitrobenzoic acid, 4 mL methanol, and 1 mL 0.2 M tris buffer (pH 8.2) and was kept at 25 °C for 15 min. The sample was centrifuged for 30 min at 1250× *g*. An aliquot of the resulting supernatant was detected at 412 nm [18]. 

For the determination of non-protein SH, homogenates were treated with TCA and centrifuged. The supernatant (100 μL) was mixed with 0.05 mL 0.01 M NaNO_2_, 0.45 mL 0.1 M H_2_SO_4_ and was permitted to stand for 5 min. The sample was added to 0.5% sulfamic acid ammonium solution (0.2 mL), 0.1 mL 1% HgCl_2_, 0.9 mL 3.4% sulfanilamide, and 1 mL 0.1% N-naphthyl ethylenediamine (in 0.4 M HCl). The mixture was kept for 5 min and was detected at 540 nm using glutathione (GSH) as a standard [19]. Catalase activity was assayed at 532 nm according to Aebi’s method [20]. Results were expressed in units/mg protein. 

### 2.8. RNA Extraction and Reverse Transcriptase-Polymerase Chain Reaction (RT-PCR)

For total RNA extraction, each frozen liver tissue (0.1 g) was homogenized in Trizol reagent (Invitrogen). Using the iScript cDNA Synthesis Kit (Bio-Rad, Hercules, CA, USA), cDNA was synthesized from 1 μg RNA samples following the manufacturer’s protocols. PCR amplification was performed in a 20 μL reaction mixture containing 1 μg of cDNA and the primer sets (Bioneer, Daejeon, Korea) (Table 1) targeting different genes using a Promega GoTaq Flexi DNA Polymerase PCR kit. PCR conditions were as follows: Initial denaturation at 95 °C for 2 min, followed by 35 cycles of denaturation at 95 °C for 30 s, annealing at 60 °C for 90 s, and extension for 72 °C for 60 s. A final extension was applied at 72 °C for 5 min. The amplified products were separated by 1.5% agarose gel electrophoresis and visualized under UV light illumination (Gel Doc/ChemiDoc imager, Azure) after ethidium bromide staining. GAPDH was used as an internal control for the normalization of mRNA levels.

### 2.9. Western Blotting Analysis

Liver tissues were homogenized and equal amounts of protein (30 μg) were separated on 7–12% sodium dodecyl sulfate-polyacrylamide gel electrophoresis (SDS-PAGE) and transferred to a polyvinylidene difluoride membrane (Millipore). The membranes were incubated in 5% nonfat milk solution for 1 h at room temperature followed by incubation with 1:1000 dilution of primary antibodies in tris-buffered saline Tween-20 (TBST) at 4 °C overnight with the following primary antibodies against total JNK, p-JNK, total Akt, p-Akt, Heme oxygenase (HO)-1, type I collagen (COL1), α-SMA, GAPDH, TFIIB, β-actin (Santa Cruz Biotechnology), and phosphatidylinositol 3-kinase (PI3K) p110 α (Cell Signaling). After washing with TBST solution, the membranes were incubated with a 1:10,000 dilution of anti-mouse, anti-goat, and anti-rabbit (Santa Cruz Biotechnology) for 1 h at room temperature. An enhanced chemiluminescence detection system determined the protein level. Western blot for GAPDH, β-actin, or TFIIB was performed to ensure equal sample loading. For evaluating activation, NF-κB and Nrf2 levels were determined in liver cytosolic or nuclear extracts, respectively. A bicinchoninic acid assay kit (Pierce, Rockford, IL, USA) was employed to measure protein concentration. 

### 2.10. Immunohistochemistry

Immunostaining was performed using the streptavidin–biotin complex (ABC) method as described in the previous report [15]. In brief, the sections were incubated for 20 min with a 0.3% H_2_O_2_ solution, then with 2% BSA, and finally for 16–18 h at 4 °C with anti-α-SMA antibody (Santa Cruz Biotechnology) in sequence. The sections were incubated for 2 h with an anti-mouse biotinylated antibody, diluted 1:200 (Jackson ImmunoResearch Laboratories, West Grove, PA, USA), and then for 1 h with an ABC reagent (Vectastain Elite kit; Vector Laboratories, Burlingame, CA, USA). Light microscopy slides were observed and photographed using an Olympus BX50 microscope.

### 2.11. Effect of Oligonol on HSC Proliferation

HSC-T6 cells, an immortalized rat HSC line, were kindly provided by Prof. S. L. Friedman (Icahn School of Medicine at Mount Sinai, New York, NY). The cells were cultured at 37 °C in a 5% CO_2_ in a DMEM medium containing 10% fetal bovine serum, 1% glutamine, 100 U/mL penicillin, and 100 U/mL streptomycin. The cells (1.0 × 10^4^ cells/well) were treated with a series of concentrations of oligonol (10, 20, 30, 50, 70, and 100 μg/mL) for 48 h, or treated with 70 μg/mL of oligonol for 0, 3, 6, 24, 36, 48, and 72 h to examine the dose- or time-dependent oligonol effect on HSC proliferation, respectively. Cell viability was assessed using MTT (3-(4,5-dimethylthiazol-2-yl)-2,5-diphenyl-tetrazolium bromides) assay.

### 2.12. Statistical Analyses

All results are expressed as the mean ± SE of the indicated number of replicates. For statistical differences, data were analyzed by one-way analysis of variance (ANOVA). A *p* value of 0.05 or less was considered statistically significant. 

## 3. Results

### 3.1. Changes in Serum Parameters in Rats Intoxicated by DMN

**Figure 2:**
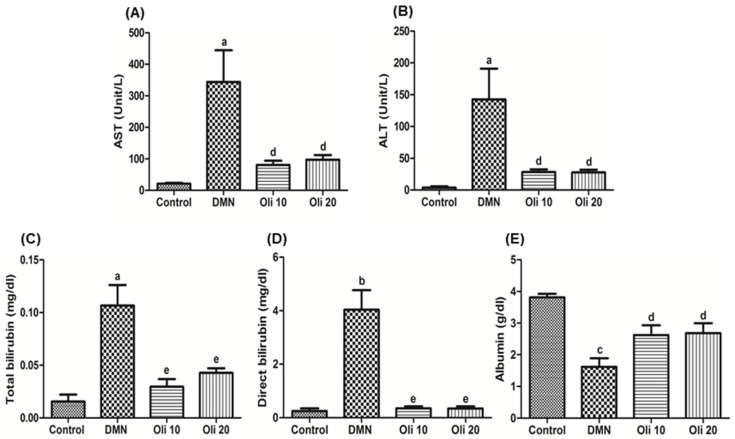
Changes in serum parameters in Rats Intoxicated by DMN. (**A**) Aspartate transaminase (AST); (**B**) Alanine transaminase (ALT); (**C**) Total bilirubin; (**D**) Direct bilirubin; (**E**) Albumin. Groups are as described in “Materials and Methods”. Values are mean ± SE of *n* = 6 rats/group. ^a^ *p* < 0.05, ^b^ *p* < 0.01, and ^c^ *p* < 0.001 vs. the control group, and ^d^ *p* < 0.05 and ^e^ *p* < 0.01 vs. the DMN group.

As shown in Figure 2, exposure to DMN resulted in liver injury as indicated by significant increases in the activities of AST, ALT (Figure 2A,B), the concentrations of total bilirubin and direct bilirubin (Figure 2C,D), and decreased albumin level indicated liver dysfunction (Figure 2E). Administration of oligonol at a dose of 10 and 20 mg/kg improved these liver biomarkers. 

### 3.2. Liver Histopathology

**Figure 3:**
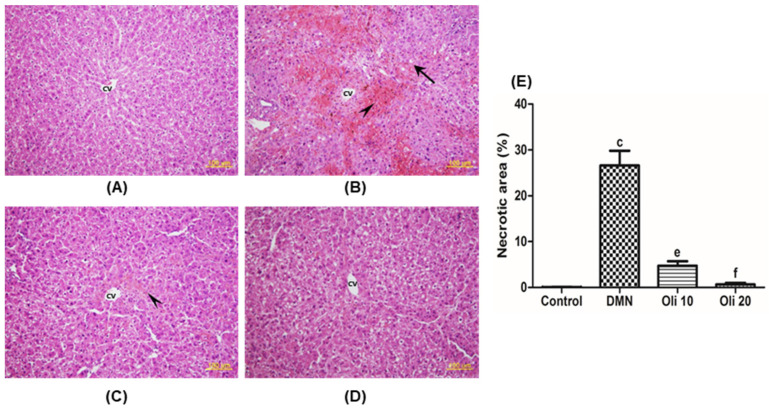
Effects of oligonol on dimethylnitrosamine (DMN)-induced histopathological changes in rat livers. H&E staining of liver sections from: (**A**) Control rats; (**B**) DMN-treated rats; (**C**) DMN-treated rats with oligonol (10 mg/kg); (**D**) DMN-treated rats with oligonol (20 mg/kg). (**E**) Quantitative analysis of necrotic area observed on H&E stained sections. ^c^ *p* < 0.001 vs. the control group, and ^e^ *p* < 0.01 and ^f^ *p* < 0.001 vs. the DMN group. CV: Central vein, arrowhead: Collagenous septa, arrows: Fatty changes. All images are original magnification ×400.

The effect of oligonol on DMN-induced histopathological changes in the liver was evaluated on H&E-stained liver sections. The control group’s liver showed normal hepatic architecture with central vein surrounded by typical radiating hepatic cords (Figure 3A). The DMN-intoxicated rat liver showed distorted tissue architecture, extensive coagulative necrosis, and loss of hepatocytes’ cell structural integrity in the centrilobular zones (Figure 3B). Notably, administration of oligonol showed an improvement of these hepatic toxicities together with normalization in liver architectures (Figure 3C,D). Quantitative analysis of necrotic area observed on H&E stained sections supported the oligonol’s hepatoprotective effect against the DMN-induced liver injury. 

### 3.3. Suppression of the DMN-Induced Oxidative Stress by Oligonol Treatment

**Figure 4:**
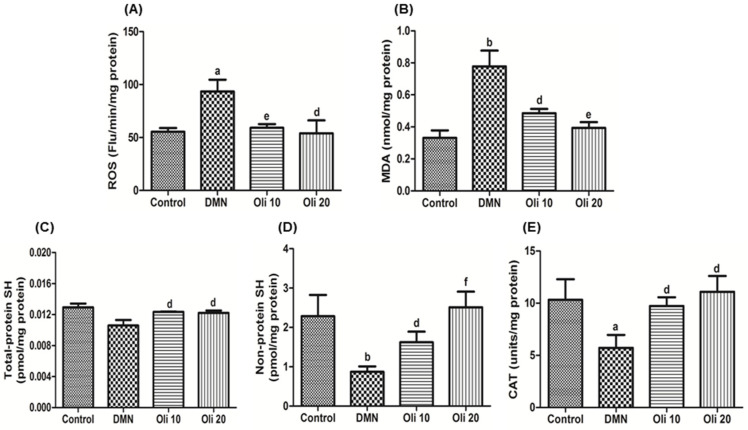
Effect of oligonol on reactive oxygen species (ROS, **A**), malondialdehyde (MDA, **B**), total SH (**C**), non-protein SH levels (**D**), and catalase activity (**E**) in rat livers intoxicated with DMN. Values are mean ± SE of *n* = 6 rats/group. ^a^ *p* < 0.05 and ^b^ *p* < 0.01 vs. the control group, and ^d^ *p* < 0.05, ^e^ *p* < 0.01, and ^f^ *p* < 0.001 vs. the DMN group.

Total ROS in the liver homogenates was measured using a DCFDA probe, and the lipid peroxidation levels were examined by measuring the lipid peroxidation product MDA. ROS levels and the MDA amounts were increased in the DMN-intoxicated rats, but they were suppressed by oligonol administration in a dose-dependent manner (Figure 4A,B). The levels of the total SH and non-protein SH (Figure 4C,D) and catalase activity, an intracellular antioxidant enzyme (Figure 4E) in liver homogenates were measured. Administration of oligonol at a dose of 10 and 20 mg/kg to DMN-intoxicated rats completely restored the suppressed antioxidant factors and brought their values near to those of the control group. 

### 3.4. Effects of Oligonol against DMN-Induced Liver Inflammation and NF-κB Activation

**Figure 5:**
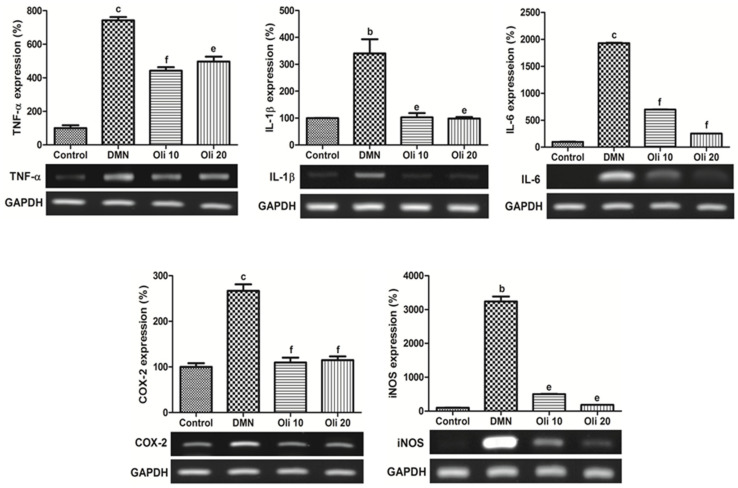
Effects of oligonol on the pro-inflammatory mediators. mRNA expression of TNF-α (**A**), IL-1β (**B**), IL-6 (**C**), COX-2(**D**), and iNOS (**E**) in rat liver intoxicated with DMN was assessed by RT-PCR. Values are mean ± SE (*n* = 3). ^b^ *p* < 0.01 and ^c^ *p* < 0.001 vs. the control group, and ^e^ *p* < 0.01 and ^f^ *p* < 0.001 vs. the DMN group.

**Figure 6:**
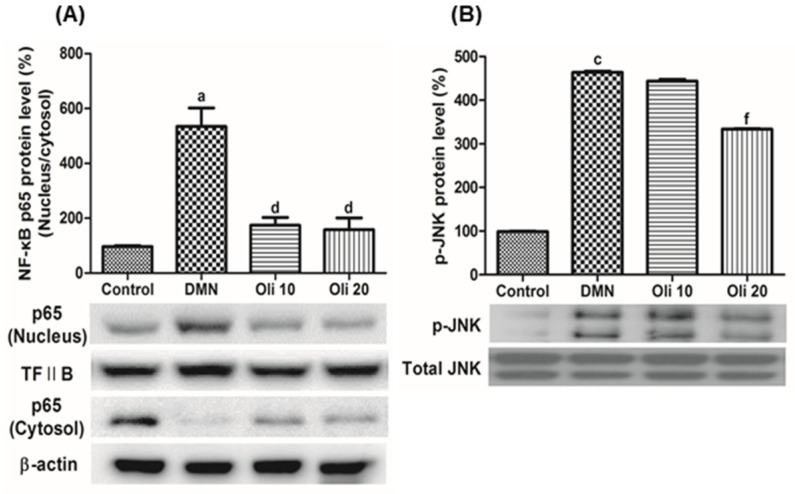
Effects of oligonol on DMN-induced NF-κB p65 activation (**A**) and JNK phosphorylation (**B**). Western blotting was performed to detect the relative level of NF-κB p65 in the nuclear and cytosol and phosphorylated JNK, and TFIIB, β-actin and total JNK were used as a loading control, respectively. Values are mean ± SE *(n* = 3). ^a^ *p* < 0.05 and ^c^ *p* < 0.001 vs. the control group, and ^d^ *p* < 0.05 and ^f^ *p* < 0.001 vs. the DMN group.

RT-PCR measured the expression of mRNA of TNF-α, IL (interleukin)-1β, IL-6, cyclooxygenase (COX)-2, and inducible nitric oxide synthase (iNOS) in the liver and quantified by normalization against the expression of the housekeeping gene GAPDH. DMN-induced elevation of these pro-inflammatory mediators’ expression was significantly suppressed in the oligonol-treated rats’ liver (Figure 5). NF-κB activation was based on the analysis of the translocation of Rel A (p65) into cell nuclei from the cytoplasm by using Western blot analysis. The level of NF-κB p65 in the nuclear and cytosol compared with TFIIB and β-actin, respectively, was quantified by image analysis. An enhancement of nuclear NF-κB and a reduction of cytosolic NF-κB was found in the rat livers by DMN treatment (Figure 6A), and oligonol treatment markedly reversed these DMN-induced phenomena. These results demonstrate that oligonol treatment in DMN-intoxicated rats strongly inhibited NF-κB activation by suppressing the translocation of NF-κB p65 from the cytosol into the nuclei. To investigate a molecular mechanism of NF-κB activation in the DMN-intoxicated rats, the levels of c-Jun N-terminal kinase (JNK) were measured by using Western blot analysis. The phosphorylation of JNK was increased in rats treated with DMN, and oligonol supplements decreased this phosphorylated JNK level (Figure 6B).

### 3.5. Effects of Oligonol on Nrf2 Activation and Akt Phosphorylation in DMN-Induced Liver Injuries

**Figure 7:**
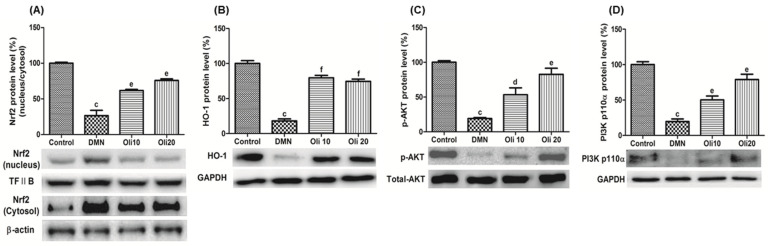
Effects of oligonol on Nrf2 activation (**A**), HO-1 production (**B**), and PI3K (**C**) and Akt activation (**D**) in DMN-intoxicated rat liver. Western blotting was performed to detect these protein levels and quantified by image analysis. The relative level of Nrf2 in the nuclear and cytosol compared with TFIIB and β-actin, respectively. The band intensity of phosphorylated Akt was normalized to total Akt. Values are mean ± SE (*n* = 3). ^c^ *p* < 0.001 vs. the control group, and ^d^ *p* < 0.05, ^e^ *p* < 0.01, and ^f^ *p* < 0.001 vs. the DMN group.

The protein levels of transcription factor Nrf2 in the nuclear and cytoplasm were also examined. Western blotting results of Nrf2 protein indicate that the ratio of Nrf2 amounts in the nuclei to the cytosol had significantly reduced in the DMN-injured rats, compared with the control animals, and the ratio was significantly increased by oligonol treatment (Figure 7A). To confirm the activation of Nrf2 in DMN-intoxicated rat liver by oligonol treatment, level of HO-1, which exists downstream of the Nrf2 and is a product of the gene, was examined. The HO-1 level in DMN-intoxicated rat livers was induced by oligonol treatment, consistent with Nrf2 activation (Figure 7B). We measured PI3K and phosphorylated Akt protein levels through Western blot analysis to investigate the molecular mechanism of Nrf2 activation by oligonol. PI3K and phosphorylated Akt protein levels were reduced in DMN-treated rats. However, oligonol treatment to DMN-intoxicated rats significantly and dose-dependently increased phosphorylated Akt and PI3K protein levels (Figure 7C,D). 

### 3.6. Antifibrotic Effect of Oligonol in DMN-Induced Chronic Liver Injuries

**Figure 8:**
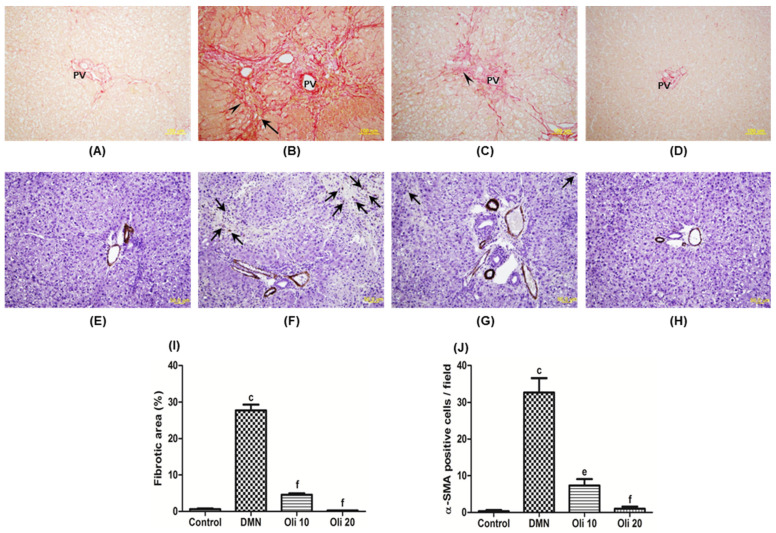
Histopathological analyses of rat liver sections using Sirius red staining (**A**–**D**) and immunohistochemistry for α-SMA (**E**–**H**). Liver samples were taken from the control (A and E), DMN group (B and F), oligonol treated groups (C and G: 10 mg/kg + DMN; D and H: 20 mg/kg + DMN). Almost no collagen fibers in the liver sections taken from the control group, except the portal area (PV). The DMN group’s liver exhibited increased collagen content (thick arrow), and fatty changes (arrowhead) (**B**), and scattered α-SMA positive cells (thin arrow) (**F**). Oligonol treatment reduced the collagen content and α-SMA positive cells and the oligonol groups show almost the same appearance as the control group. (**I**) Quantification of fibrotic area observed on the Sirius red stained section. (**J**) Number of α-SMA positive cells per field. All images are original magnification ×400. ^c^ *p* < 0.001 vs. the control group, and ^e^ *p* < 0.01 and ^f^ *p* < 0.001 vs. the DMN group.

Collagen accumulation (stained in red) was examined from the Sirius red staining. Except for the portal area, there were almost no collagen fibers in the liver sections of the control group (Figure 8A), whereas the DMN group exhibited increased collagen content (Figure 8B) in the liver tissue. However, the collagen content markedly returned to normal level dose-dependently in the oligonol groups (Oli10 and Oli20) (Figure 8C,D). Quantitative analysis of fibrotic area observed on Sirius red stained sections supported the oligonol’s anti-fibrotic effect against the DMN-induced liver injury (Figure 8I). 

**Figure 9:**
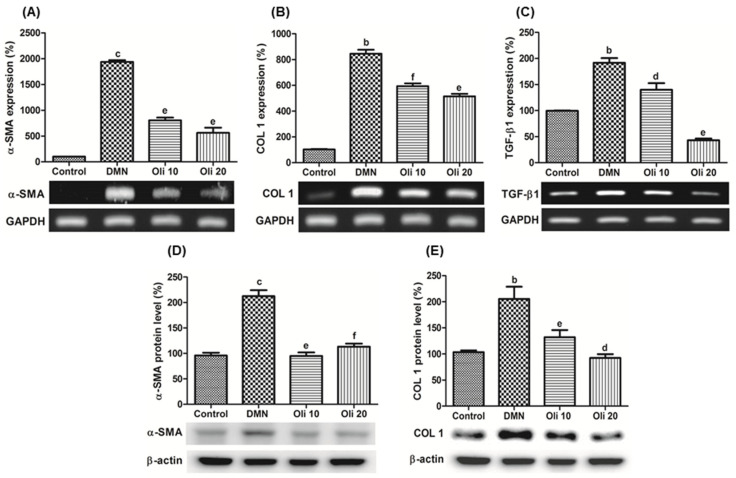
Effects of oligonol on the profibrogenic factors in the rat livers. RT-PCR was performed to measure mRNA expression of α-SMA (**A**), COL1 (**B**), and TGF-β1 (**C**). Western blot analysis was performed to measure α-SMA (**D**) and COL1 (**E**) protein levels. Values are mean ± SE (*n* = 3). ^b^ *p* < 0.01 and ^c^*p* < 0.001 vs. the control group, and ^d^ *p* < 0.05, ^e^ *p* < 0.01, and ^f^ *p* < 0.001 vs. the DMN group.

The α-SMA is a specific marker for activated HSCs. Immunohistochemistry results showed that the number of α-SMA-positive cells in the periportal fibrotic band areas of the DMN-treated rats (Figure 8F) was reduced by oligonol treatment, almost to the same level as that observed in the liver of the control group (Figure 8G,H). These results indicate that oligonol treatment prevents DMN-induced liver fibrosis via inhibiting the HSC activation. The level of α-SMA and COL1 was assessed to evaluate oligonol’s effect on DMN-induced liver fibrosis at the mRNA (Figure 9A,B) and protein levels (Figure 9D,E). α-SMA and COL1 expressions were markedly increased in the DMN group, whereas the expression was significantly reduced in oligonol-treated groups. The fibrogenic cytokine TGF-β1 mRNA expression was reduced by oligonol treatment in DMN-intoxicated rats (Figure 9C).

### 3.7. The Effects of Oligonol on HSC-T6 Cells

**Figure 10:**
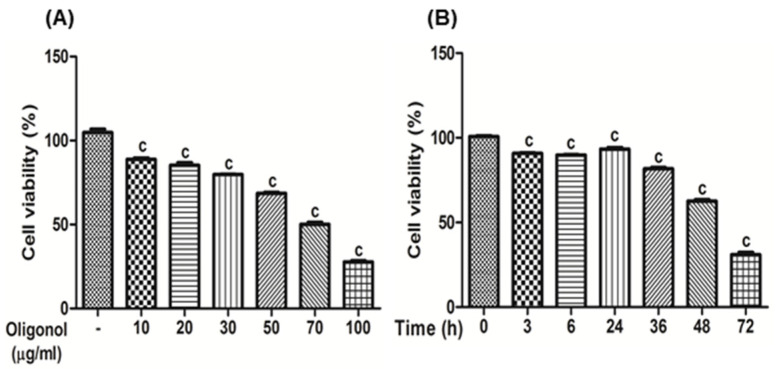
Oligonol suppresses hepatic stellate (HSC)-T6 cell proliferation. Cells (1 × 10^4^/well) in a 96-well plate were allowed to adhere overnight, and then the culture medium was replaced with fresh, 10% serum DMEM. (**A**) HSC-T6 cells were treated with a series of concentrations of oligonol for 48 h. (**B**) HSC-T6 cells were treated with 70 μg/mL of oligonol for 0, 3, 6, 24, 36, 48, and 72 h, and cell viability estimated by the MTT assay is expressed as a percentage based on the oligonol-untreated cells. Values are the mean ± SE (*n* = 3). ^c^ *p* < 0.001 vs. the control group.

The effects of oligonol on HSC-T6 cells’ viability were determined by MTT assay. Oligonol treatment significantly inhibited the proliferation of HSC-T6 cells in a dose- (Figure 10A) and time-dependent manner (Figure 10B), respectively. 

## 4. Discussion

Chronic liver injuries lead to hepatic fibrogenesis if the resultant hepatocellular necrosis and inflammation is not interrupted by a proper cellular response or medical intervention. DMN is a potent hepatotoxin, mutagen, and carcinogen. Reactive metabolites generated from its metabolism by the cytochrome P4502E1 are known as the ultimate toxicants to DMN-induced hepatic toxicity [21]. Repeated low doses of DMN in rats lead to hepatic necrosis, chronic inflammation, liver fibrosis, or liver cancer. DMN-induced liver fibrosis models have various advantages, such as progressive and prominent pathological changes, high fibrosis reproduction rates, and relatively low mortality in experimental animals [22]. We reported the antioxidative, anti-inflammatory, and hepatoprotective properties of oligonol on CCl_4_-induced acute liver injury model in rats in the previous study [14]. Here, we examined whether oligonol exerts an anti-fibrotic effect on the DMN-induced chronic liver injury model in rats. 

In this study, intoxication with DMN in rats for four weeks had induced liver injury and dysfunction, which was assessed by serum parameters and liver histopathology from H&E staining. However, oligonol’s treatment ameliorated these alterations. It is reported that hepatocyte injury is followed by inflammation and increased cytokines such as TNF-α, IL-1β, and IL-6. Due to these cytokines’ uncontrolled and harmful action, many investigations of inflammatory organ injuries have focused on these cytokines [23]. Considerable evidence supports that TNF-α and IL-1β are related to liver diseases’ pathogenesis by activating the NF-κB signaling pathway [24]. NF-κB family consists of five members RelA (p65), RelB, c-Rel, p50, and p52. In addition to RelA subunit, c-Rel is also reported to play a role in liver fibrosis development [25,26]. However, we focused on the oligonol’s effect on RelA activation in our present study. Oligonol’s anti-inflammatory effect was investigated by analyzing the quantities of NF-κB p65, TNF-α, IL-1β, and IL-6. Our data showed that NF-κB activation and the resultant increases of TNF-α, IL-1β, and IL-6 mRNA expression in DMN-induced liver injury were inhibited by oligonol treatment. Besides pro-inflammatory cytokines, NF-κB regulates pro-inflammatory COX-2 and iNOS production. In this study, COX-2 and iNOS mRNA expression was also significantly increased in the DMN group’s liver, and oligonol supplements reduced these increased inflammatory protein production. These data suggest that oligonol exerts an anti-inflammatory effect by inhibiting NF-κB activation, suppressing pro-inflammatory mediators’ production. 

MAPK pathway is a significant signal transduction pathway associated with cell proliferation, survival, death, differentiation, and transformation [27]. Diverse extracellular and intracellular stimuli activate the MAPK pathways. MAPK transmits stimuli-induced signals to the downstream NF-κB, which upregulates target genes such as TNF-α, IL-1β, IL-6, COX-2, and iNOS. Our results also showed that phosphorylation of JNK, which is one of the MAPK family members, is related to DMN-induced hepatic injuries in rats, and oligonol inhibited the DMN-induced JNK activation. These results implied that oligonol exerts its anti-inflammatory effect on the DMN-intoxicated rat liver via inhibiting the JNK/NF-κB signaling pathway. 

It is reported that events involved in the metabolic activation and detoxification process of DMN result in oxidative stress that further contributes to liver damage [28]. In this study, overall oxidative status and total ROS production in the liver were significantly enhanced, and the levels of the total SH and non-protein SH were reduced in DMN-intoxicated rats. Induction of lipid peroxidation, related to the balance between oxidative stress and antioxidative defense system, increased in the DMN models. Oligonol improved oxidative status in DMN-intoxicated chronic liver injuries in rats, which is consistent with the results of oligonol’s antioxidative properties against lipid peroxidation and ROS production in the acute liver injury induced by CCl_4_ [14]. Nrf2 and its downstream proteins are critical in protecting cells from oxidative stress- and chemical-induced damage to the liver [29]. Binding Nrf2 to the antioxidant response element (ARE) on DNA regulates more than 100 genes, involving HO-1 and intracellular antioxidant enzyme catalase, leading to cellular protection. The critical role of Nrf2 in adapting to toxicity is confirmed in Nrf2 knockout mice that are more sensitive to many toxicants, including the hepatotoxicity of acetaminophen [30] and the pulmonary toxicity of cigarette smoke [31]. There is much evidence that the activation of Nrf2 can be an effective strategy against the drug- or xenobiotic-induced liver toxicity. An abundant number of natural products exhibited hepatoprotective effects via the Nrf2 signaling pathway [32]. In this study, oligonol treatment increased Nrf2 activation with enhanced HO-1 expression level and catalase activity in DMN-intoxicated rats. Cellular GSH level might be relevant to up-regulation of the expression of GSH-related defense enzymes in the hepatocyte, such as GSH synthase and GSH peroxidase, which exist downstream of Nrf2 and contribute to the protection of cells from oxidative stress. Improved oxidative status in DMN-treated rats by oligonol treatment may result from Nrf2 activation along with the antioxidative effect of oligonol. PI3K/Akt pathway is reported to play critical roles in regulating Nrf2 and ARE-dependent protection against oxidative stress by increasing intracellular GSH synthesis and its concentration [33]. In this study, PI3K and phosphorylated Akt levels were increased by oligonol treatment in the DMN-intoxicated rats. The results suggest that activation of Nrf2 in DMN treated rats might be relevant to PI3K activation, which subsequently catalyzes the Akt phosphorylation. 

Repeated inflammation of hepatocytes is known to lead to liver fibrosis, and in this study, a 4-week-treatment of DMN produced fibrotic liver, assessed from an evaluation with an SR staining and type I collagen expression. Central process in the liver fibrosis is related to HSC activation initiated by products formed during liver injuries. Kupffer cells can release ROS and pro-inflammatory cytokines during liver damages, thereby recruiting more inflammatory cells and aggravates the injury and oxidative stress. HSC activation is also driven by the crucial mediator cytokine TGF-β1, leading to excessive ECM protein production such as type I collagen [34]. In this study, the increased levels of TGF-β1 and type I collagen in DMN-treated rats were suppressed by oligonol. Expression of α-SMA, which is a hallmark of activated HSCs, was increased and the oligonol treatment suppressed the HSC activation, as assessed by reduced α-SMA mRNA expression and its protein production, and α-SMA positive cell numbers. To provide further evidence of oligonol’s anti-fibrotic properties, oligonol’s effects on HSCs were examined using HSC-T6 cells. Oligonol suppressed the proliferation of HSC-T6 cells time- and dose-dependently. These results indicate that oligonol exerts anti-fibrotic activity, in part, by inhibiting HSC proliferation. 

In this experiment, we assessed the oligonol’s protective effect on the hepatic injury. To evaluate whether oligonol exhibits a therapeutic effect against liver fibrosis, additional research in pre-established liver injury is needed. Repeated liver inflammation leads to liver fibrosis, liver cirrhosis, and liver cancer. Since oligonol has shown anti-inflammatory effects against acute and chronic liver injury models induced by CCl_4_ and DMN, respectively, oligonol can be a potential supplements for anti-cancer therapy. Therefore, further study is required to evaluate oligonol’s effect against liver cancer models induced by hepatotoxins such as diethylnitrosamine (DEN).

## 5. Conclusions

The present study’s findings indicate that oligonol is highly effective in preventing DMN-induced chronic liver damage, which is most likely mediated by its activity to suppress oxidative stress and lipid peroxidation as an antioxidant. It has the anti-inflammatory capacity to inhibit JNK/NF-κB pathway to induce the pro-inflammatory mediators such as TNF-α, IL-1β, IL-6, COX-2, and iNOS. Oligonol activated Nrf2 and increased the production of HO-1 and PI3K, and induced Akt phosphorylation, suggesting that PI3K/Akt/Nrf2 signaling pathway is related to the improved oxidative stress condition of oligonol treated rat livers. Oligonol exhibited anti-fibrotic properties against DMN-induced rat liver fibrosis. Anti-fibrotic activity of oligonol is most likely due to its capacity to suppress the HSC activation by lessening hepatic inflammation via inhibition of NF-κB activation in the early stages of liver fibrosis and restoring the balance of oxidative status via Nrf2 activation, which prevents lipid peroxidation and ROS production. Therefore, these findings suggest that oligonol may potentially be a therapeutic agent to prevent hepatic inflammation and its development into hepatic fibrosis.

## Figures and Tables

**Table 1:** Oligonucleotide sequences used in RT-PCR analysis.

Gene		Sequences	Gene Access Number
TNF-α	F	TTCTGTCTACTGAACTTGGGGGTGATCGGTCC	XM_032888689.1
	R	GTATGAGATAGCAAATCGGCTGACGGTGTGGG	
IL-1β	F	ATGGCAACTGTTCCTGAACTCAACT	NM_031512.2
	R	CAGGACAGGTATAGATTCTTTCCTTT	
IL-6	F	CGAGCCCACCAGGAACGAAAGTC	M26745.1
	R	CTGGCTGGAAGTCTCTTGCGGAG	
iNOS	F	GATTCAGTGGTCCAACCTGCA	L12562.1
	R	CGACCTGATGTTGCCACTGTT	
COX-2	F	CCAGAGCAGAGAGATGAAATACCA	NM_017232.3
	R	GCAGGGCGGGATACAGTTC	
TGF-β1	F	TATAGCAACAATTCCTGGCG	NM_021578.2
	R	TGCTGTCACAGGAGCAGTG	
α-SMA	F	CCGAGATCTCACCGACTACC	XM_032891814.1
	R	TCCAGAGCGACATAGCACAG	
COL1	F	AAGAAGGCGGCAAAGGTC	XM_005257059.4
	R	GGACCTTGTTTGCCAGGT	
GAPDH	F	GACAACTTTGGCATCGTGGA	NM_017008.4
	R	ATGCAGGGATGATGTTCTGG	

F: Forward; R: Reverse; TNF-α: Tumor necrosis factor-alpha; IL-1β: Interleukin-1beta; COX-2: Cyclooxygenase-2; iNOS: Inducible nitric oxide synthase; TGF-β1: Transforming growth factor-beta1; α-SMA: Alpha-Smooth muscle actin; COL1: Type 1 collagen; GAPDH: Glyceraldehyde-3-phosphate dehydrogenase.

## Data Availability

The authors confirm that the data supporting the findings of this study are available within the article.
